# 926. COVID-19 Infections After SARS-CoV-2 Vaccination in Solid Organ Transplant Recipients

**DOI:** 10.1093/ofid/ofab466.1121

**Published:** 2021-12-04

**Authors:** Kapil Saharia, Judy Streit, Susan E Beekmann, Philip M Polgreen, Matthew Kuehnert, Dorry Segev, John W Baddley, Rachel Miller

**Affiliations:** 1 University of Maryland School of Medicine, Baltimore, MD; 2 The University of Iowa Carver College of Medicine, Iowa City, IA; 3 University of Iowa Carver College of Medicine, Iowa City, IA; 5 Musculoskeletal Transplant Foundation; Hackensack Meridian School of Medicine, Edison, NJ; 6 Johns Hopkins, Baltimore, MD; 7 Duke University, Durham, NC

## Abstract

**Background:**

Solid organ transplant recipients (SOTR) have lower humoral responses following SARS-CoV-2 vaccination. Whether this equates to reduced vaccine effectiveness in SOTR or impacts disease severity is not yet known. We used the IDSA Emerging Infections Network (EIN) to identify SARS-CoV-2 cases in vaccinated SOTR. We describe their clinical characteristics and outcomes.

**Methods:**

On 4/7/21, we requested case reports via the EIN listserv of COVID-19 infection following SARS-CoV-2 vaccination in immunocompromised individuals. Case reports were collected until June 7^th^. Online data collection included patient demographics, dates of SARS-CoV-2 vaccine administration and clinical data related to COVID-19 infection. We performed a descriptive analysis of these patient factors and compared differences between early onset (< / = 21 days after completing vaccine series) and late onset infection ( > 21 days after completing vaccine series).

**Results:**

As of 6/7/21, 34 cases of COVID-19 infection after vaccination in SOTR were submitted. Most cases (79%) occurred in individuals who were fully vaccinated. Only 3 cases (8.5%) occurred in SOTR within their first year after transplantation. Clinical characteristics are listed in Table 1. The vaccine administration date was known for 26 SOTR among whom symptoms occurred a median of 26.5 days (IQR 21.75 days, range 5-79 days) after completing the COVID-19 vaccine series. Twenty-three SOTR (68%) required hospitalization of which 12 had critical illness. Outcome data was available for 29 individuals of whom 20 (69%) demonstrated improvement. When comparing SOTR with early versus late onset COVID-19 infection in relation to vaccination timing, there were no differences in disease severity (80% vs 75% with severe or critical disease, p=NS) or outcome (30% vs 31% died or deteriorating, p=NS).

Table 1: Characteristics of Solid Organ Transplant Recipients with COVID-19 Infection Following SARS-CoV-2 Vaccination

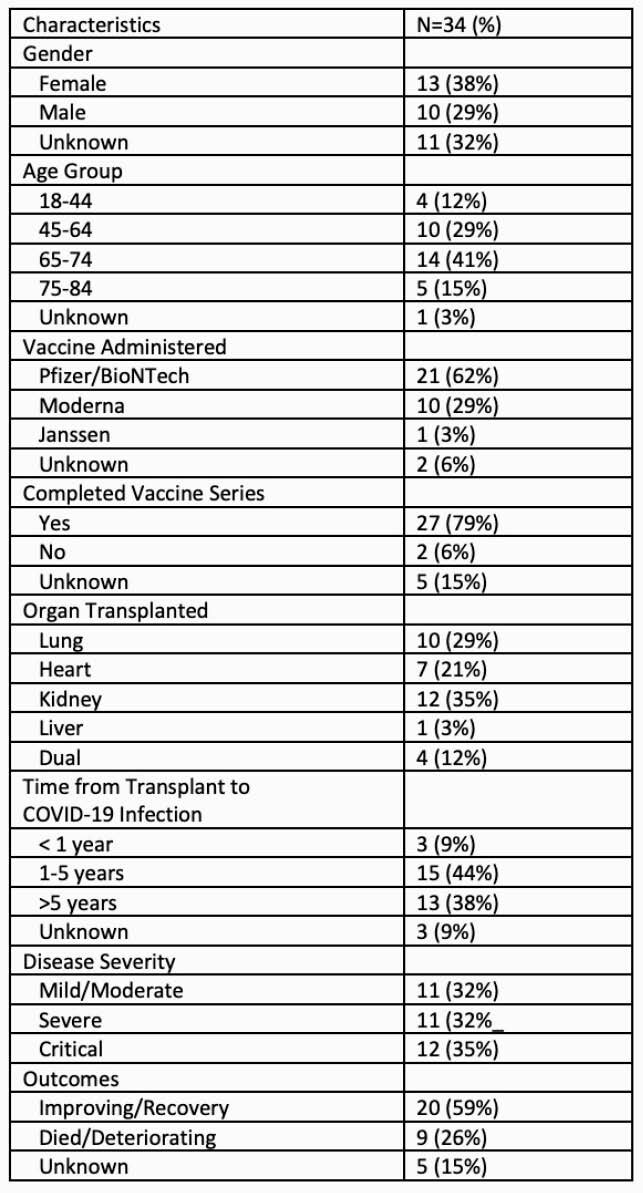

**Conclusion:**

SARS-CoV-2 infections after vaccination are occurring in SOTR, including cases of critical illness, suggesting reduced vaccine effectiveness within this vulnerable population. We did not appreciate any correlation between time from vaccination and COVID-19 disease severity or outcome. Further studies evaluating the true incidence of and risk factors for breakthrough infections among vaccinated SOTR are needed.

**Disclosures:**

**Matthew Kuehnert, M.D.**, **American Association of Tissue Banks** (Board Member)**ICCBBA** (Board Member)**Musculoskeletal Transplant Foundation** (Employee) **John W. Baddley, M.D.**, **Eli Lilly** (Consultant)**Pfizer** (Consultant)**R-Pharm** (Consultant)**Viela Bio** (Consultant)

